# Incorporating repeated measurements into prediction models in the critical care setting: a framework, systematic review and meta-analysis

**DOI:** 10.1186/s12874-019-0847-0

**Published:** 2019-10-26

**Authors:** Joost D. J. Plate, Rutger R. van de Leur, Luke P. H. Leenen, Falco Hietbrink, Linda M. Peelen, M. J. C. Eijkemans

**Affiliations:** 1 Division of Surgery, University Medical Centre Utrecht, Heidelberglaan 100, Utrecht, 3584 CX the Netherlands; 2 Julius Center for Health Sciences and Primary Care, University Medical Center Utrecht, Utrecht, the Netherlands; 3 Departments of Anesthesiology and Intensive Care Medicine, University Medical Center Utrecht, Utrecht, the Netherlands

## Abstract

**Background:**

The incorporation of repeated measurements into multivariable prediction research may greatly enhance predictive performance. However, the methodological possibilities vary widely and a structured overview of the possible and utilized approaches lacks. Therefore, we [1] propose a structured framework for these approaches, [2] determine what methods are currently used to incorporate repeated measurements in prediction research in the critical care setting and, where possible, [3] assess the added discriminative value of incorporating repeated measurements.

**Methods:**

The proposed framework consists of three domains: the observation window (static or dynamic), the processing of the raw data (raw data modelling, feature extraction and reduction) and the type of modelling. A systematic review was performed to identify studies which incorporate repeated measurements to predict (e.g. mortality) in the critical care setting. The within-study difference in c-statistics between models with versus without repeated measurements were obtained and pooled in a meta-analysis.

**Results:**

From the 2618 studies found, 29 studies incorporated multiple repeated measurements. The annual number of studies with repeated measurements increased from 2.8/year (2000–2005) to 16.0/year (2016–2018). The majority of studies that incorporated repeated measurements for prediction research used a dynamic observation window, and extracted features directly from the data. Differences in c statistics ranged from − 0.048 to 0.217 in favour of models that utilize repeated measurements.

**Conclusions:**

Repeated measurements are increasingly common to predict events in the critical care domain, but their incorporation is lagging. A framework of possible approaches could aid researchers to optimize future prediction models.

## Background

To achieve the maximum predictive performance, the choice of the underlying statistical model is essential [1]. Conventional methods, e.g. linear or logistic regression analysis, have been successfully utilized in prediction models. However, the increasing computational power *and* the growing availability of big data facilitates the use of more powerful and advanced methods [2, 3]. This may be particularly of importance when repeated measurements of the predictor variables, i.e. sequential or temporal data, yield additional prognostic value.

Advanced methods to handle these repeated measurements in prediction research have arisen from two different research fields, namely statistics and informatics. Statistically, methods come from a mathematical basis and usually explicitly model the associations between predictor(s) and outcome, whereas the research field of informatics and machine learning often utilizes trial-and-error training processes to implicitly model (all) possible associations [4]. The rapid emergence of machine learning models has resulted in a seemingly endless wealth of models, approaches and accompanying names. In the ongoing search to optimize predictions and incorporate them in a useful clinical tool, a structured overview could be of great help to direct further research.

Therefore, this study provides an overview of the currently utilized approaches to incorporate repeated measurements for multivariable prediction within the setting of critical care. This setting is chosen because the constant monitoring of critically ill patients leads to wealthy amounts of sequential data, which could well be utilized to timely predict and thereby identify clinical deterioration. This allows for timely, potentially life-saving interventions.

More specifically, we (1) propose a framework for the possible approaches to incorporate repeated measurements in prediction research in the critical care setting, (2) determine what methods are currently used and (3) assess the added prognostic performance of these methods as compared to methods which do not incorporate repeated measurements.

## Methods

**Fig. 1:**
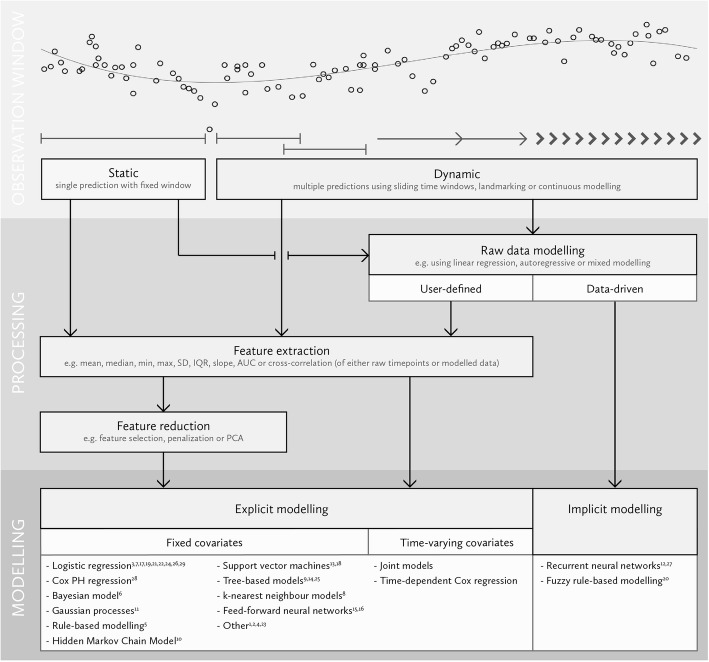
Proposed framework for the sequential steps in the incorporation of repeated measurements in multivariable prediction. This Figure shows the proposed framework in which approaches and steps to incorporate repeated measurements in prediction research are shown. The framework consists of three domains: the observation window used to make predictions (static or dynamic), the processing of the raw data (raw data modelling, user-defined or data-driven, feature extraction and feature reduction) and explicit or implicit modeling using fixed or time-varying covariates

First, the different approaches and steps to incorporate repeated measurements of the independent variables are graphically visualized in the proposed framework (Fig. 1). This framework was based on the authors’ and several expert opinions. The proposed framework consists of three domains: (A) the observation window used, (B) the data processing phase and (C) the modelling phase in which predictions are made.

### Proposed framework – observation window

The o*bservation window* is defined as the time window of measurements which are used to make predictions. Depending on the aim of the study, a *static* or *dynamic* observation window can be used. Static refers to a fixed time window to make a single prediction, whereas the dynamic observation window refers to the use of multiple observation windows for multiple time-varying predictions, i.e. repeated predictions at multiple time points. An example of the use of a static observation window can be found in the prediction of (in-hospital) mortality using the first 24 h of collected physiological data at the Intensive Care Unit (ICU) [5, 6].

Dynamic observation windows are often used for different prediction aims, such as the timely recognition of an adverse hypotensive event *during* ICU admission [7]. This requires multiple predictions at different time points. The actual observation window used for these dynamic predictions differs per study, probably according to the clinically hypothesized value of the repeated measurements for the prediction purpose at hand. If it is expected that only data from the last 6 h before the time of prediction is relevant to predict the outcome, a sliding time-window may be used, in which only the measurements 6-h prior to the prediction time are used [8]. However, if one would reason that all cumulative data is important to predict ICU-mortality, all available aggregate data at each time point could be used. The situation in which multiple models are fit at various ‘landmark times’ using all available information up to the time of prediction, is also commonly referred to as landmark models [9]. Another possibility is to use a continuous modelling strategy that uses every timepoint and outputs a prediction at every timepoint as well.

### Proposed framework – processing phase

For the processing phase, three steps can be distinguished. First, the raw data can be modelled before features are extracted for prediction. Possibilities include the fitting of linear functions, such as linear regression or linear mixed models, and non-linear functions, such as splines.

The second step is the extraction of (summary) features from the raw data, such as the mean, standard deviation and the skewness. These features try to capture certain time characteristics in a summary measure of the data. Alternatively, features can be extracted from the raw data modelling step, if this has been performed. For example, from a mixed model the estimated value and the slope at a certain time point can be extracted for use in a Cox proportional hazards model, as is simultaneously done in joint models [10]. Also, new features could be obtained from either previously extracted features or the raw data, e.g. correlation measures [11].

A third step is the reduction of the numbers of features to be used in the model, in order to prevent overfitting or reduce the computational burden of the model. This can be done in multiple ways, using methods such as penalized regression or principal component analysis.

### Proposed framework – modelling phase

The eventual modelling can be achieved through the use of either two-step or end-to-end modelling, wherein two-step refers to the use of user-defined raw data representations and predictors and/or time-based correlations as explicit input for the prediction model. The two-step models have been further divided into models which use fixed covariates, such as logistic regression, and models which allow the covariates to change over time (time-dependent Cox regression or joint models) [10, 12]. The models with fixed covariates can also be fit multiple times (e.g. in time-slicing/landmarking), but then at each prediction time the fixed covariates are modelled again in a different risk set, as opposed to allowing the covariates to change within the model [8]. End-to-end modelling is distinguished from two-step modelling in that the model uses only the raw timepoint as an input and jointly optimises both pattern discovery and prediction internally [13]. An example of this is the recurrent neural network, a deep learning network which uses loops to pass information from one step to another in the network [14].

### Systematic review - identifying currently used methods

To identify currently used methods to incorporate repeated measurements in multivariable prediction research in the critical care setting, we subsequently performed a systematic review. The protocol for this systematic review can be found at PROSPERO (protocol number: CRD42018093978).

### Systematic review - information sources

Two independent researchers (JP and RL) performed a comprehensive literature search in multiple electronic databases (Medline and Embase). All publications up to 23.05.2018 were searched. The following keywords were used: (“repeated measurements” AND “Prediction” AND “critical care unit”) AND “Predictive performance”, and synonyms and Mesh Terms of those [see Additional file 1 for all search terms]. Disagreements were resolved via consensus or consultation of a third independent reviewer (RE). All full texts were screened by one author (JP), while a random subset (10%) was assessed by another author (RL): Cohen’s kappa’s coefficient was obtained to measure the inter-rater agreement.

### Systematic review - study selection

As inclusion criteria for full text review the following terms were used: (1) published in English or Dutch and (2) reporting upon multiple measurements per patient at the ICU (or Intermediate Care Unit (IMCU) or cardiologic care unit). Excluded were articles which used repeated measurements to predict prediction errors of a diagnostic device (e.g. in glucose monitoring), or to predict the response (rise in blood pressure) of a fluid bolus challenge in (hypovolemic) patients or focused on post-surgery patients only. The rationale for these exclusions is that these studies are more on the diagnosis domain (diagnostic advice, fluid response yes/no) or resemble a specific condition entity (post-surgery) as opposed to general critical care admissions.

Studies which did report to have used repeated measurements, but did not incorporate these repeated measurements into their prediction model were denoted as ‘single-timepoint models’, e.g. models that used only the last available measurement, and excluded during full text review. Studies that incorporated only one repeatedly measured predictor were also excluded. The rationale for the exclusion of the latter studies was that univariable prediction reflects a different prediction problem, in which usually the possible predictive value of one predictor (e.g. a biomarker) is assessed. This stands in contrast with the multivariable prediction problem, where as much information is used as possible to optimize the predictions. Also, conference abstracts were excluded if an accompanying conference paper could not be found at the electronical databases or via Google and Google Scholar.

Studies were included in the meta-analysis (1) if they compared an analysis which incorporated repeated measurements with a single-timepoint regression analysis, i.e. an analysis in which they did not incorporate repeated measurements, (2) if a c-statistic could be obtained and (3) if the results were internally validated (i.e. with bootstrapping, k-fold cross validation or a split-sample approach).

### Systematic review - data collection

The data from all articles was extracted using a standardized data extraction form. Data collected were: year of publication, inclusion criteria, sample size used, determinants measured at baseline, determinants repeatedly measured, primary outcome definition, number of events (if dichotomous outcome), statistical analyses performed, internal validation methods, reported performance measures, and the performed comparative statistical analyses, if applicable.

If the primary analysis resulted in multiple similar performance measures (e.g. c-statistic at different prediction times), the optimal measurement was extracted as we hypothesized that articles which did report only a single performance measure were also likely to report their optimal performance [15]. As reported performance measures, we chose to extract the c-statistic (discrimination) and observed to expected ratio (O:E ratio, calibration) [16]. If these measures were not reported, we tried to obtain these performance measures, using related measures as described by Debray et al. [16].

### Systematic review - risk of bias

The risk of bias of prognostic modelling studies could be assessed using the Prediction model Risk Of Bias Assessment Tool (PROBAST), which is a tool to assess the risk of bias and applicability concerns of prediction model studies [17]. However, this was not done here because the comparison of interest (i.e. models with versus without repeated measurements) is methodological, and focuses on the analytical comparison *within* studies. Furthermore, the PROBAST statement is designed for clinical studies and thus focuses on assessing the risk of bias for the *intended use* and *target population* of a model, both issues which are not very relevant for this study. However, to reduce the bias in included studies, we chose to exclude studies which did not internally validate their prediction models.

### Systematic review - summary measures

The main measures of interest were the discriminative performance and the calibration of the model, although measures concerning the calibration (e.g. O:E ratio or calibration slope) were too rarely reported to be used in this study. Therefore, we only obtained summary measures of the discriminative performance (c-statistic). The c-statistic is similar to the area under the receiver operating curve, which is a graphical illustration of the false positive rate versus the true positive rate at each possible threshold. It can be interpreted as the probability that a random diseased subject is correctly rated with greater suspicion than a random non-diseased subject [18].

If uncertainty around the c-statistic was not reported, this was approximated with the following formula:

$$ Var\left( logit(c)\right)\approx \frac{\left[1+{s}^{\ast}\frac{1-c}{2-c}+{t}^{\ast}\frac{c}{1+c}\right]}{\mathrm{stc}\left(1-\mathrm{c}\right)}, $$

where s is the number of observed events, t is the total of non-events, and ${s}^{\ast }={t}^{\ast }=\frac{1}{2\left(s+t\right)}-1$ [16, 19].

To assess the change in c statistic due to the incorporation of repeated measurements in the analysis, the within-study difference in c-statistics was required. The mean change was simply obtained by subtracting the mean of the single-timepoint model from the mean of the repeated measurements model. However, the variance of this change depends upon the variances of the c-statistics of both the models *and* the covariance of their related c-statistics.

$$ Var(diff)= Var(c1)+ Var(c2)-2\ast Cov\ \left(c1,c2\right) $$

where diff is the mean difference, c1 is the c-statistic of the 1st (repeated measurements) model and c2 the c-statistic of the 2nd (single-timepoint) model [20].

As this covariance (or the very similar correlation) between these c-statistics is not reported in current articles and, to our knowledge, has not been studied before, this covariance was estimated using a simulation on a previously published dataset in the critical care setting [21]. In this simulation, the resulting covariance between the bootstrapped (*n* = 200) c-statistic from a single-timepoint model (5 variables at time of prediction) and the c-statistic from a repeated measurements model (same variables plus their means over the entire observation window) was 0.0097 (correlation 0.72). More detail with respect to this simulation can be found in Additional file 2.

### Meta-analysis - synthesis of results

Although summary measures of within-study differences could theoretically be pooled to assess the added prognostic performance of incorporating repeated measurements in multivariable predictions, we chose not to do to this for two reasons. First, the predicted outcome differs, which means that the possible achievable c-statistic differs per study. Therefore, for some studied outcomes it will be difficult if not impossible to increase the c-statistic with the incorporation of repeated measurements. Second, the statistical heterogeneity between the studies, measured with the I [2, 22], was too high to warrant pooling of the results. The I [2] describes the percentage of variation across studies that is due to heterogeneity rather than chance, and can be calculated as follows:

$$ {I}^2=100\%x\ \left(Q- df\right)/Q $$

where Q is Cochran’s heterogeneity statistic and df the degrees of freedom [22]. Cochran’s heterogeneity statistic can be calculated as follows:

$$ T=k\left(k-1\right)\frac{\sum_{j=1}^k{\left({X}_{\circ j}-\frac{N}{k}\right)}^2}{\sum_{i=1}^b{X}_{i\circ}\Big(k-{X}_{i\circ \Big)}} $$

where *k* is number of treatments, *X*_∘*j*_ is the column total for the j^th^ treatment, b is the number of blocks, *X*_*i*∘_ is the row total for the i^th^ block, N is the grand total [23].

All statistical analyses were performed using R software for statistical computing version 3.3.2 [24]. with the additional packages “metamisc” [25] and “forestplot” [26]. Where applicable, the reporting of this article follows the Preferred Reporting Items for Systematic Reviews and Meta-Analyses (PRISMA) statement (Additional file 3) [27].

## Results

### Study selection

**Fig. 2:**
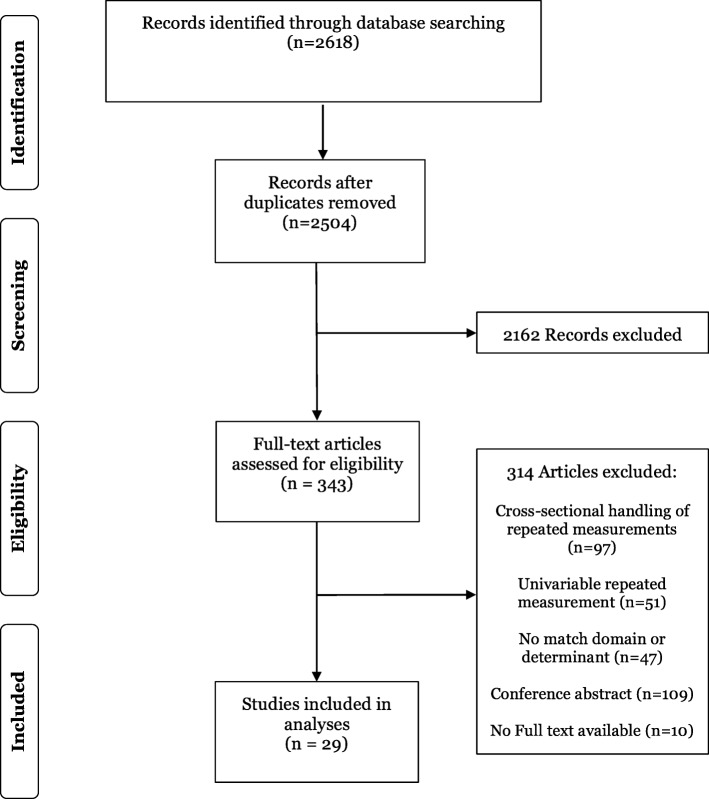
Preferred Reporting Items for Systematic Reviews and Meta-Analyses (PRISMA) flow diagram for study selection [27]

From 2618 titles and abstracts, 343 articles were selected for full-text review (Fig. 2). Upon full text screening, there were 47 studies which did not match domain or determinant: although these studies should have been excluded in the title/abstract screening, we were not always able to obtain the required information (e.g. repeated measurements or not) from the title/abstract screening.

Of all eligible full text articles which reported on having repeated measurements (*n* = 177), 97 (54.8%) studies did not incorporate these repeated measurements in their prediction models. From the 80 other studies, 51 (63.4%) studies only reported having one repeatedly measured predictor, while 29 studies incorporated multiple repeated measurements. Cohen’s kappa coefficient for the full text sample screening was 0.76, while no extra studies were included after joint discussion of the disagreements.

### Study characteristics

**Fig. 3:**
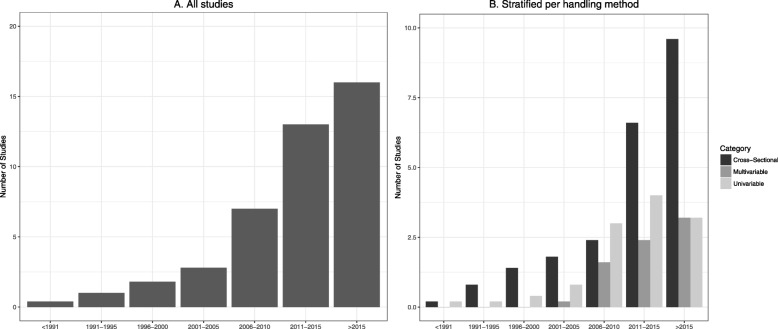
Annual number of studies with repeated measurements. This Figure shows the annual number of studies with reported measurements in the critical care setting. Figure **a** depicts annual averages of all studies and Figure **b** depicts annual averages of the studies per type of analysis performed, in which single-timepoint studies do not incorporate the repeated measurements, univariable studies incorporate just one repeatedly measured variable and the included studies incorporate repeated measurements of multiple variables

An overview of the study characteristics can be found in Additional file 4. Figure 3 shows the annual number of all studies with repeated measurements in the critical care setting. This includes all studies which mentioned the use of repeated measurements (i.e. studies with single-timepoint models (*n* = 97), univariable repeated measurements studies (*n* = 51) and the multivariable repeated measurements studies (*n* = 29)).

The annual number of all studies which mentioned the use of repeated measurements increased from 2.8/year (2001–2005) to 16.0/year (> 2015). The annual number of studies which did not incorporate these measurements in their analysis (single-timepoint studies) increased from 1.8/year (2001–2005) to 9.6/year (> 2015). The annual number of studies that did incorporate these measurements in their multivariable analysis increased from 0.2/year (2001–2005) to 3.2/year (> 2015). The annual number of studies that incorporated a single repeatedly measured predictor in their analysis increased from 0.8/year (2001–2005) 3.2/year (< 2015).

The outcome of interest in the majority (*n* = 10) of studies was hospital- or ICU mortality [28–37]. Other outcomes were the occurrence of sepsis [38, 39] or septic shock [40], the need for dialysis [41], the need for specific clinical interventions (e.g. vasopressor use) [42], transfer out of the ICU [11], transfer from the IMCU to the ICU [43], spontaneous breathing after breathing trial [44], recovery status (clinically assessed) [45], acute hypotensive event [7, 46, 47], cardiac arrest [48], long-term neurological outcome [49–51], length of ICU stay [8], delayed cerebral ischemia [52], depressed left-ventricular ejection fraction [53].

This Figure shows the annual number of studies with reported measurements in the critical care setting. A depicts annual averages of all studies and B depicts annual averages of the studies per type of analysis performed, in which single-timepoint studies do not incorporate the repeated measurements, univariable studies incorporate just one repeatedly measured variable and the included studies incorporate repeated measurements of multiple variables.

### Overview of approaches to include repeated measurements

From this, it follows that 9 studies used a static observation window and 20 studies used dynamic observation windows. The raw data was modelled in 5 studies, with either autoregressive modelling [42, 44, 45] or linear regression [7, 47].

The most frequently mentioned extracted features were the mean (*n* = 11) [7, 11, 28, 32, 34, 37, 38, 47, 48, 52, 53], median (*n* = 5) [7, 37, 43, 47, 49], standard deviation or variance (*n* = 4) [7, 30, 37, 47], maximum (*n* = 4) [30, 31, 34, 43],, linear regression slope (*n* = 3) [7, 47, 48], delta change (*n* = 3) [11, 35, 38], skewness and kurtosis (*n* = 3) [7, 37, 47], minimum (*n* = 2) [34, 43], interquartile range (*n* = 2) [7, 47] and variability [49, 52]. Several other features were also mentioned by the included studies, such as intervals [49], frequency-domain analysis [49], correlations [49], relative energy [7, 47] and in a study using EEG data alpha-to-delta, signal power, Shannon entropy, delta coherence, regularity, number of bursts/min and burst correlation [51].

To extract features which describe the relation between already extracted features, various methods were described: multidimensional correlation analysis [11, 38], association-rule mining [29], sequence patterns of categorized variables [32, 36, 40], convolutional dictionary learning [52], the ratio between means in sequential periods [48], the number and duration of categorical variables under/above a pre-defined threshold [8, 33, 50] and cross-correlation patterns between multiple repeated measurement trends [7, 47].

Feature reduction is most often realized through univariable selection (also referred to as ‘univariate’ selection), which statistically tests the relationship between one independent variable and the outcome, e.g. by the student’s t test (*n* = 10) [11, 28, 35, 38, 44]. Various other methods have also been described (*n* = 6) [7, 29, 40, 47, 48, 52].

Lastly, a vast array of two-step modelling methods and three end-to-end modelling methods have been described: the long short-term recurrent neural network [39], the echo-state network [41] and fuzzy rule-based modelling [53]. Of the two-step models, the logistic regression (*n* = 8) [8, 28, 33, 35, 36, 43, 44, 50] was most frequently used. No two-step models with time-varying covariates, e.g. time-varying Cox regression and joint models, were utilized in this clinical setting. It should be noted that the choice of the eventual model is also dependent upon the type of outcome (e.g. binary, time to event or continuous).

### Application of the model

As an example, Cancio et al. assessed the value of arterial blood gas (ABG) in the prediction of mortality after burn injury, using data (*n* = 162) collected during the first 2 days of admission [28]. They used a static observation window and extracted the mean of all measured ABG values. Further, reduced their features via univariable selection and, finally, applied logistic regression. This framework shows the arbitrary decisions they made in each step. Some decisions may be fixed to answer their research question (static observation window) or fixed due to the data (small sample should lead to two-step modelling). Decisions in other steps though, such as the use of different summary features (e.g. *change* in ABG values) and another (or no) feature reduction method, could have improved the predictive value of their model.

On the other hand, Kam et al. sought to *timely* predict sepsis in intensive care unit patients, utilizing a long short-term recurrent neural network [39]. Due to this research question and sufficient data they were able to choose for a dynamic (almost continuous modelling) observation window and end-to-end modelling. This framework shows that, aside from the choice of dynamic window (e.g. 3-h, measurements per minute) and the specifications of the LSTM RNN no other arbitrary choices need to be made.

### Performance of analyses with and without repeated measurements

**Fig. 4:**
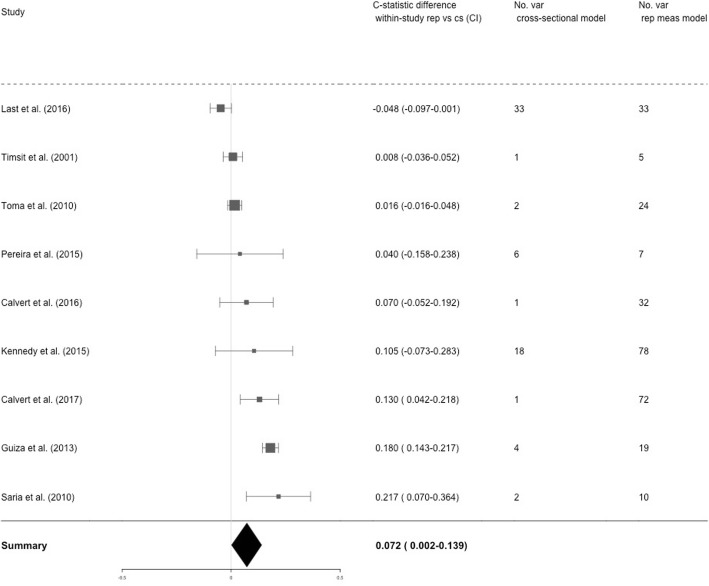
Comparison between analyses which do and do not include repeated measurements. This Figure shows the difference in within-study c-statistics (confidence interval) of studies which reported analyses both with and without the incorporation of repeated measurements. Abbreviations: rep = repeated measurements analysis; cs = single-timepoint analysis; no. var. = number of variables in the model

The within-study differences in c-statistics (and their confidence interval) of studies which reported upon analyses with and without incorporating repeated measurements are shown in Fig. 4. In all studies, single-timepoint logistic regression was used as a comparison analysis. Furthermore, internal validation was performed via cross-validation [37, 38, 49, 50, 53], bootstrapping [35, 36] or split-sample [11, 37, 39, 48]. One study was excluded from this analysis, as it did not use any validation method [42]. The statistical heterogeneity, as measured by the I [2], was 87.73%. No summary measures were obtained due to reasons outlined in the methods section.

## Discussion

This study illustrates that repeated measurements are increasingly common in the critical care setting, although their incorporation in prediction modelling is lagging. To provide insight into the use of repeated measurements, a structured overview of possible and currently utilized approaches is provided. This framework could aid researchers with repeated measurements data in their decision-making to optimize future prediction models, as this likely increases the discriminative performance of the models.

These results are in line with a recent systematic review on electronical health care records, which also found that the amount of studies with repeated measurements is increasing [15]. This same study also supports the finding that the majority of studies do not incorporate the longitudinal aspect of their data (61%) [15]. Possible reasons for the failure to implement repeated measurements could be (1) the increased complexity of methods which incorporate repeated measurements, (2) uncertainty about their potential benefits, (3) the decreased interpretability of such methods or (4) the lack of added clinical value of complex models, because these models cannot easily be implemented into clinical practice.

Our study extends the current knowledge of repeated measurements approaches by providing a structured overview of the possible and the currently utilized methods in the critical care setting. Although multiple comparisons of methodological approaches have been performed, these (primary) studies have all used arbitrarily chosen methods as opposed to a structured literature review [54, 55]. However, this framework is based upon experts’ opinion as opposed to an overall consensus, which means that its structure may be debatable. Even so, the rapid increase in use of methods to incorporate repeated measurements asks for a harmonization of nomenclature.

From the currently used approaches it follows that the focus in the critical care setting is mainly on prediction with a dynamic time window, in which features are extracted and used in fixed-covariate models. This may be due to acquaintance with such (commonly used) models, as this group of models is also widely used in situations without repeated measurements. A very similar emphasis on these (fixed covariate) models was observed in the Physionet challenge 2012 on ICU-mortality prediction [56].

The utility of the proposed framework lies in the categorization in the different steps of the (desired) approaches towards incorporating repeated measurements. This provides valuable insights into the vast array of possible approaches and methodologies, facilitates in choosing the approach and helps in the comparison between different approaches and models. Therein it can aid medical, epidemiological, artificial intelligence engineers and statistical researchers who wish to perform predictions with repeated measurements or aid those who read and assess such studies. Which approach is the best, is very dependent upon the data and research question at hand, and should be analysed through internal (or external) validation.

Further research should focus on the ongoing comparison of the different types of approaches to incorporate repeated measurements, through comparative cohort- or simulation studies using different models and reporting on both the discrimination and calibration. The step thereafter would be to see whether the implementation of such models truly increases patient outcome. Also, a more comprehensive simulation study would enhance our knowledge and understanding of the correlation between models with repeated and without repeated measurements.

This study has multiple strengths. First of all, it is (to our knowledge) the first to present a structured overview of possible and utilized approaches to incorporate repeated measurements. Also, its focus is on the critical care domain, a clinical domain where a wealth of repeatedly measured data from electronical health care systems and monitors is available, with the potential to truly support decision-making and increase patient safety [57]. Therefore it is likely that most modelling advancements will initially be developed and implemented in this setting, which makes the identification of the current focus in this setting highly relevant.

The limitations of this study are that the proposed structured framework is based upon experts’ opinion as opposed to an overall consensus. Further, the critical care domain is only one clinically domain where repeated measurements are used and therefore we might have missed (novel) methods in other domains, particularly those which use less-frequently sampled data, e.g. annually collected data from multiple clinical visits [58]. However, we have no reason to assume that the framework reported here would not apply to this kind of data. Another important limitation is that the focus of the literature search was on studies which reported measures of discrimination or calibration in their abstract, while these measures are not necessarily mentioned in the abstracts of studies which utilize repeated measurements for prediction purposes in this setting. This may mean that our findings are not comprehensive, but it seems unlikely that the approaches in the studies which do not report the calibration or discrimination fall outside this proposed framework.

## Conclusions

Repeated measurements are increasingly common to predict events in the critical care domain, but their incorporation in current prediction models is lagging. Therefore, a framework of possible and currently utilized approaches is provided. This could aid researchers with repeated measurements data to optimize future prediction models and thereby improve patient outcome.

## Supplementary information

**Additional file 1.** Search terms used in the systematic review.

**Additional file 2.** Performed simulation study to obtain an estimate of the covariance between the c-statistic for a single timepoint model and the c-statistic of the repeated measurements model.

**Additional file 3.** PRISMA checklist.

**Additional file 4.** Characteristics of included studies.

## Data Availability

All data are present in the tables, figures and appendices.

## Abbreviations

ABG: Arterial Blood Gas
ICU: Intensive Care Unit
IMCU: Intermediate Care Unit;
O:E-ratio: Observed to Expected ratio
PRISMA: Preferred Reporting Items for Systematic Reviews and Meta-Analyses
PROBAST: Prediction model Risk Of Bias Assessment Tool
