# Supplementary material for: Incorporating repeated measurements into prediction models in the critical care setting: a framework, systematic review and meta-analysis
Source: BMC Med Res Methodol. 2019 Oct 26;19:199. doi: 10.1186/s12874-019-0847-0 (PMC6815391; doi:10.1186/s12874-019-0847-0)
Supplement: Supplementary file 1 — Additional file 1. Search terms used in the systematic review. [file 12874_2019_847_MOESM1_ESM.pdf]

Pubmed

Number	Search terms	hits
1: methodologic domain	((repeated[tiab] OR repeatedly[tiab] OR sequential[tiab] OR longitudinal[tiab] OR consecutive[tiab] OR temporal[tiab] OR time[tiab]) AND (measurements[tiab] OR measured[tiab] OR data[tiab] OR sequence[tiab] OR series[tiab])) OR “longitudinal study”[tiab] OR “longitudinal cohort”[tiab]	1,332,643
2: methodologic domain	("prediction"[tiab] OR "predict"[tiab] OR "prognostic study" OR "prognostic research"[tiab] OR "prognosis"[tiab] OR (risk[tiab] AND event[tiab]) OR (probability[tiab] AND event[tiab]) OR (occurence[tiab] AND event[tiab]) OR (risk[tiab] AND outcome[tiab]) OR (probability[tiab] AND outcome[tiab]) OR (occurence[tiab] AND outcome[tiab]) OR "decision support"[Title/Abstract] OR prognosis[Majr])	993,903
3: clinical domain	"intensive care"[tiab] OR "critical care"[tiab] OR "intensive care units"[Mesh] OR "critical care"[Mesh] OR "intermediate care"[tiab] OR "high dependency care"[tiab] OR "step-down"[tiab] OR "step down"[tiab] OR "medium care"[tiab] OR "progressive care"[tiab] OR "cardiologic care unit"[tiab] OR "mimic"	252,287
4: outcome	(discrimination[Title/Abstract] OR discriminate[tiab] OR calibration[Title/Abstract] OR calibrate[Title/Abstract] OR sensitivity[Title/Abstract] OR specificity[Title/Abstract] OR “false positive”[tiab] OR “false negative”[tiab] OR auc[Title/Abstract] OR "area under the curve"[Title/Abstract] OR "c-statistic"[Title/Abstract] OR "c statistic"[Title/Abstract] OR concordance[Title/Abstract] OR ROC[Title/Abstract] OR "receiver operating curve"[Title/Abstract] OR “O:E ratio”[Title/Abstract] OR "observed to expected ratio"[Title/Abstract] OR “discriminative performance”[tiab] OR “prediction error”[tiab] OR “R squared”[tiab] OR “coefficient of determination”[tiab] OR accuracy[tiab] OR predictive value of tests[Majr])	1,189,877
4	#1 AND #2 AND #3 AND #4	1083

Embase

Number	Search terms	hits
1: methodologic domain	((repeated:ab,ti OR repeatedly:ab,ti OR sequential:ab,ti OR longitudinal:ab,ti OR consecutive:ab,ti OR temporal:ab,ti OR time:ab,ti) AND (measurements:ab,ti OR measured:ab,ti OR data:ab,ti OR sequence:ab,ti OR series:ab,ti)) OR ‘longitudinal study’:ab,ti OR ‘longitudinal cohort’:ab,ti OR 'longitudinal studies'/mj	1,867,761
2: methodologic domain	'prediction':ab,ti OR 'predict':ab,ti OR 'prognostic study' OR 'prognostic research':ab,ti OR 'prognosis':ab,ti OR (risk:ab,ti AND event:ab,ti) OR (probability:ab,ti AND event:ab,ti) OR (occurence:ab,ti AND event:ab,ti) OR (risk:ab,ti AND outcome:ab,ti) OR (probability:ab,ti AND outcome:ab,ti) OR (occurence:ab,ti AND outcome:ab,ti) OR 'decision support':ab,ti OR 'prognosis'/mj	1,360,699
3: clinical domain	'intensive care':ab,ti OR 'critical care':ab,ti OR 'intensive care units'/exp OR 'critical care'/exp OR 'intermediate care':ab,ti OR 'high dependency care':ab,ti OR 'step-down':ab,ti OR 'step down':ab,ti OR 'medium care':ab,ti OR 'progressive care':ab,ti OR 'cardiologic care unit':ab,ti OR 'mimic'	832,778
4: outcome	discrimination:ab,ti OR discriminate:ab,ti OR calibration:ab,ti OR calibrate:ab,ti OR sensitivity:ab,ti OR specificity:ab,ti OR ‘false positive’:ab,ti OR ‘false negative’:ab,ti OR auc:ab,ti OR ‘area under the curve’:ab,ti OR ‘c-statistic’:ab,ti OR ‘c statistic’:ab,ti OR concordance:ab,ti OR ROC:ab,ti OR ‘receiver operating curve’:ab,ti OR ‘O:E ratio’:ab,ti OR ‘observed to expected ratio’:ab,ti OR ‘discriminative performance’:ab,ti OR ‘prediction error’:ab,ti OR ‘R squared’:ab,ti OR ‘coefficient of determination’:ab,ti OR accuracy:ab,ti OR ‘predictive value of tests’/mj	1,817,089
5: combined	#1 AND #2 AND #3 AND #4 AND [embase]/lim NOT ([embase]/lim AND [medline]/lim)	1,889
