# Supplementary material for: Incorporating repeated measurements into prediction models in the critical care setting: a framework, systematic review and meta-analysis
Source: BMC Med Res Methodol. 2019 Oct 26;19:199. doi: 10.1186/s12874-019-0847-0 (PMC6815391; doi:10.1186/s12874-019-0847-0)
Supplement: Supplementary file 2 — Additional file 2. Performed simulation study to obtain an estimate of the covariance between the c-statistic for a single timepoint model and the c-statistic of the repeated measurements model. [file 12874_2019_847_MOESM2_ESM.docx]

The aim of this simulation was to estimate the degree of correlation and covariance between the c-statistics of analyses which do and which do not utilize repeated measurements using data from a critical care setting

The following steps were taken:

1. Loading data from a previous report.^1^ This data is collected at the surgical Intermediate Care Unit, a unit which is logistically situated between the hospital ward and the Intensive Care Unit. The data includes repeated measurements of the following 5 variables, collected by the nurses from the electronical health care data every 0.1 day (2.4 hours, mean value over period).

The data is represented as one row per measurement time, with the following variables in the columns:

- Administered oxygen (liters/min)
- Respiratory rate (breaths/min)
- Heart rate (pulse/min)
- Mean arterial pressure (mmHg)
- Noradrenalin use (ug/kg/min).

The outcome was the occurrence of an event (transfer to the Intensive Care Unit) within 12 hours (yes/no).

2. Compute the mean over the first 24 hours per variable.

3. Only keep measurements within 0.9 and 1 day after and measurements without any missing values (complete case analysis)

4. Fit logistic regression model using all cross-sectional measurements (model 1, cs) and using all cross-sectional measurements *and* mean values (model 2, rep)

5. Fit both models on bootstrapping sample (n=200) and obtain both c-statistics per sample

6. Obtain correlation and covariance between the c-statistics of both samples

**References**

1. Plate JD, Peelen LM, Leenen LL, Hietbrink F. Validation of the VitalPAC Early Warning Score at the Intermediate Care Unit. *World journal of critical care medicine*. 2018;7(3):39-45.
