# Supplementary material for: Incorporating repeated measurements into prediction models in the critical care setting: a framework, systematic review and meta-analysis
Source: BMC Med Res Methodol. 2019 Oct 26;19:199. doi: 10.1186/s12874-019-0847-0 (PMC6815391; doi:10.1186/s12874-019-0847-0)
Supplement: Supplementary file 4 — Additional file 4. Characteristics of included studies. [file 12874_2019_847_MOESM4_ESM.docx]

| **ID** |  |  |  | **Database Specification** | | | | | | | | **Statistical Performance** | | | | | | | |
| --- | --- | --- | --- | --- | --- | --- | --- | --- | --- | --- | --- | --- | --- | --- | --- | --- | --- | --- | --- |
|  | **Study (year)** | **Country** | **MIMIC-II** | **Included patients** | **No. of patients** | **Variables used** | **Meas interval (freq data)** | **Pred. time** | **Dyn?** | **Event** (pred horizon) | **No. of events** | **Validation method** | **Statistical Analyses 1** | **c-statistic** | **O:E-ratio** | **Statistical Analysis 2** | **c-statistic** | **O:E-ratio** |  |
| 1 | Calvert et al. (2017)^1^ | USA | Y | Adults >48h | 4.000 | Physiologic | 1 hour | 2 days | N | Patient stability (yes/no) | 191 | Split-sample (50%, 92 events) | 1. Multidimensional Analysis for Correlations using raw and delta value per hour  2. Univariable selection (if significant correlation)  3. Linear predictor of weights based on univariable correlation | 0.81 | *NR* | Logistic regression (cross-sectional) | 0.68 | *NR* |  |
| 2 | Calvert et al. (2016)^2^ | USA | Y | Adults w/o SIRS at admission | 1.394 | Physiologic, laboratory | 5-hour window | 0 | Y | Sepsis diagnosis (3h before) | 40 | Cross-validation (*4*-fold) | 1. Summary measures (Mean and delta) of past 5-h meas window. Delta classified as pos, negligible, neg and correlation between meas (doublets and triplets) obtained.  2. Linear predictor: coefficients optimized via *optimization technique* | 0.92 | 0.43 | Logistic regression (cross-sectional) | 0.85 | *NR* |  |
| 3 | Cancio et al. (2016)^3^ | USA | N | Adults with burn injury | 162 | Physiologic, laboratory | *NR* | 2 days | N | Mortality (NS) | 55 | NA | 1. Summary measures (mean arterial blood gas measures)  2. Univariable selection  3. Logistic regression | 0.826 | *NR* | NA | NA | NA |  |
| 4 | Chaparro et al. (2011)^4^ | Spain | N | Patients on spontaneous breathing trial | 153 | Respiratory patterns | 5 min | 2 days | N | Spontaneous breathing (48h) | 38 | Cross-validation (leave-one-out) | 1. Autoregressive modelling of respiratory patterns  2. Univariable selection of relevant parameters  3. Classification model | *NR^1^* | *NR* | Linear discriminant analysis and SVM | *NR* | *NR* |  |
| 5 | Cheng and Wang (2015)^5^ | USA | N | Children <21 years old | 4.975 | Laboratory | *irr* | 2 days | N | Mortality (6h) | *NR* | Cross-validation (*5*-fold) | 1. Association rule mining  2. Causality-based Rule Selection provides probability based on average of top number of rules (12) | *NR* | *NR* | Top-confidence-based rule selection | *NR* | *NR* |  |
| 6 | Crump et al. (2009)^6^ | USA | N | Adults >48h | 52 | Physiologic | 4 hours | 2 days | N | Recovery status (bad/good) | 6 | Split-sample (n=16, 2 events) | 1. Standard moving average to predict physiological trends  2. Bayesian network model using raw vital sign values and predicted personal trend | 0.857 | *NR^2^* | NA | *NR* | *NR* |  |
| 7 | Cuthbertson et al. (2007)^7^* | UK | N | HDU patients | 136 | Physiologic | NS | *var* | N | ICU transfer (48h) | 67 | NA | 1. Summary measures (median, minimum, maximum) of trends  2. logistic regression | 0.90 | *NR^2^* | NA | *NR* | *NR* |  |
| 8 | Ebadollahi et al. (2010)^8^ | USA | Y | *NR* | 1.500 | Physiologic | 1-min (2-hour window) | *NS* | Y | Adverse hypotensive event (1h) | 590 | Split-sample (20%) | 1. Features extracted from trends  2. Match to similar patients (*k-*most) based on clinical labels.  3. Predicting future trend based on linear regression of previous trend and *k*-similar trends | *NR^2^* | *NR* | Mean | *NR* | *NR* |  |
| 9 | Ghose et al. (2015)^9^ | Australia | Y | Adults >48h | 4.000 | Physiologic and laboratory | 2 hours | 2 days | N | Mortality (in-hospital) |  | Cross-validation (*10*-fold) | 1. Summary measures (mean, max, standard deviation)  2. Classification with random forest | 0.83 | *NR* | Logistic regression (cross-sectional) | *NR* | *NR* |  |
| 10 | Ghosh et al. (2017)^10^ | Australia | Y | Adults | 1.310 | Physiologic | 1 hour | *NS* | Y | Septic shock (30 min) | 209 | Cross-validation (*5*-fold) | 1. Sequential patterns extracted from repeated measurements and discretized (e.g. 6-6-6-5-7)  2. Selection of sequential patterns using a *Lexicographic Sequence Tree*  3. Hidden Markov Chain model to capture interactions between patterns | *NR^2^* | *NR* | SVM | *NR^2^* | *NR* |  |
| 11 | Guiza et al. (2013)^11^ | Belgium | N | Traumatic Brain Injury patients | 160 | Physiologic | 4 hours (24h observation) | 4 hours | Y | Poor neurological outcome (6 months) | 29 | Cross-validation (*5*-fold) | 1. Features extracted from trends (median, variability, intervals, frequency-domain analysis, correlations)  2. Classification using Gaussian Processes | 0.90 (CI 0.87-0.92) | ∂ | Logistic regression (cross-sectional) | 0.72 (CI 0.69-0.75) | ∂ |  |
| 12 | Kam and Kim. (2017)^12^ | South Korea | Y | Adults, SIRS <1h of ICU admission | 720 | Physiologic and laboratory | 1 hour | 5 hours | Y | Sepsis (3 hours) | 360 | Split-sample (20% test) | Long short-term recurrent neural network learns features by itself | 0.929 |  | Insight model (Calvert 2009), with trend data | 0.83 |  |  |
| 13 | Kennedy et al. (2015)^13^ | USA | N | Children <18 years | 212 | Physiologic | 5 – 60 min | 12 hours | Y | Cardiac arrest (1 hour) | 109 | Split-sample (33% test) | 1. Summary measures extracted (mean, slope, ratio of means between epochs)  2. Univariable selection using SVM weighting and recursive feature elimination  3. Classification using Support Vector Machines (SVM) | 0.975 |  | Logistic regression (cross-sectional) | 0.87 |  |  |
| 14 | Last et al. (2016)^14^ | Israel | N | Adults | 3.452 | Physiologic, laboratory | *var* | 1 day | Y | ICU-mortality (yes/no) | 718 | Split-sample (33% test) | 1. Summary measures obtained (daily max)  2. Sequential read-once decision tree | 0.650 | *NR* | Logistic regression (cross-sectional) | 0.698 | *NR* |  |
| 15 | Lee and Mark. (2011)^15^ | USA | Y | *NR* | 1.357 | Physiologic | 1 min | 30 minutes | Y | Acute hypotensive event (1-4h) | *NR* | Cross-validation (*5*-fold) | 1. Summary measures obtained (mean, median, SD, variance, IQR, skewness, kurtosis, linear regression slope, relative energie, cross-correlation between time series)  3. Feature reduction using principal component analysis (PCA)  2. Classification using feed-forward neural networks | 0.921 (CI 0.91-0.94) | *NR* | NA | *NR* | *NR* |  |
| 16 | Lee and Mark. (2010)^16^ | USA | Y | Adults | 1.311 | Physiologic | 1 min | 30 min |  | Acute hypotensive event (1h) | *NR* | Cross-validation (*5*-fold) | 1. Summary measures obtained (mean, median, SD, variance, IQR, skewness, kurtosis, linear regression slope, relative energie, cross-correlation between time series)  3. Feature reduction using principal component analysis (PCA)  2. Classification using feed-forward neural networks | 0.914 (CI 0.88-0.95) | *NR* | NA | *NR* | *NR* |  |
| 17 | Levin et al. (2012)^17^ | USA | N | Children <18years | 2.062 | Clinical orders | 6 hours | 6 hours | Y | Length of ICU-stay (up till 3 days) | NA | NA | 1. Summary measures (number of orders 6-h period)  2. Classification using cross-sectional logistic regression  3. Repeated per 6-h period | *NR^2^* | *NR* | NA | NA | NA |  |
| 18 | Megjhani et al. (2018)^18^ | USA | N | Subarachnoid hemorrhage patients | 488 | Physiologic | 1-250 min | 4 days | N | Delayed Cerebral Ischemia | *NR* | Cross-validation (*8*-fold) | 1. Summary measures (mean) per time period (1-240 min)  2. Extract temporal patterns using convolutional dictionary learning  3. Feature reduction using minimum redundancy maximal relevance  4. Classification using SVM | 0.78 (95% CI, 0.63–0.92) | *NR* | Logistic regression (cross-sectional) | 0.49 (0.37–0.61) | *NR* |  |
| 19 | Minne et al. (2013)^19^ | Leiden | N | Adults | 912 | SOFA, expert opinion | 1 day | 2-7 days | Y | Mortality (in-hospital) | 175 | NA | 1. Extract temporal patterns in SOFA (discretized in Low, Medium, High patterns, e.g. L, H) and summary measures (mean)  2. Classification using cross-sectional logistic regression  3. Repeated per 24-h | 0.83 | *NR^1^* | Decision tree | 0.79 | *NR^1^* |  |
| 20 | Pereira et al. (2015)^20^ | Portugal | Y | Adults | 936 | Physiologic | 25 min | 6 hours | Y | Severely depressed left ventricular ejection fraction | 115 | Cross-validation (*5*-fold) | 1. Summary measures obtained (mean)  2. Classification using fuzzy rule-based modeling: rules are constructed from data, providing weighted output | 0.71 (CI 0.57, 0.85) | *NR* | Logistic regression (mean) | 0.67 (CI 0.53, 0.81) | *NR* |  |
| 21 | Rivera-Fernández et al. (2012)^21^ | Spain | N | *NR* | 17.022 | Physiologic | 1 day | 1 day | N | Mortality (in-hospital) | 3510 | Cross-validation (*10*-fold) | 1. Use threshold to obtain number and duration of critical events  2. Classification using logistic regression | 0.833 (CI 0.823, 0.838) | *NR* | NA | NA | NA |  |
| 22 | Saria et al. (2010)^22^ | USA | N | Neonates <2000g <34 weeks | 138 | Physiologic | *var* | 3 hours | N | Long-term neurologic development | 35 | Cross-validation (*leave-one-out*) | 1. Summary measures (number of orders 6-h period)  2. Classification using cross-sectional logistic regression | 0.9151 | *NR* | Logistic regression (cross-sectional) | 0.6978 | *NR* |  |
| 23 | Stein et al. (2012)^23^ | USA | N | Comatose adults | 52 | Physiologic and brain measurements | 6 sec | 12 hours | Y | Mortality (in-hospital) | 6 | Cross-validation (*leave-one-out*) | 1. Summary measures obtained (max, min, mean, duration beneath threshold)  2. Univariable selection    2. Classification using multiple methods (compound covariate predictor, linear discriminant analysis, one-nearest-neighbor classifier, three-nearest neighbor classifier, nearest-centroid classifier, SVMs) | *NR^2^* | *NR^2^* | NA | NA | NA |  |
| 24 | Timsit et al. (2001)^24^ | France | N | Adults >3 days | 1205 | SOFA and organ dysfunction score | 1 day | 3 days | N | Mortality (in-hospital) | 57 | Bootstrapping (500 rep) (also split-sample set) | 1. Summary measures obtained (delta)  2. Univariable selection  3. Classification using logistic regression | 0.794 (CI 0.76- 0.820) | *NR* | Logistic regression (cross-sectional) | 0.786 (CI 0.757-0.812) | *NR* |  |
| 25 | Tjepkema et al. (2017)^25^ | The Netherlands | N | Adults after cardiac arrest | 283 | EEG features | 5-min | 12 hours | N | Cerebral performance (180 days) | *NR* | Split-sample (33% test) | 1. Summary measures obtained from signal (alpha-to-delta ratio, signal power, Shannon entropy, delta coherence, regularity, number of bursts/min, mean burst correlation, maximal burst correlation, and fraction of burst correlation >0.8)  2. Classification using random forest | 0.93 | *NR* | Logistic regression (cross-sectional) | 0.74 | *NR* |  |
| 26 | Toma et al. (2010)^26^ | The Netherlands | N | Adults (excluding post cardiac surgery) | 2928 | Organ dysfunction scores | 1 day | 2 days | Y | Mortality (in-hospital) | 728 | Bootstrapping (300 rep) | 1. Pattern discovery using median thresholds (e.g. 1-1-0-0-1)  2. Classification using logistic regression | 0.742 | *NR* | Logistic regression (cross-sectional) | 0.726 | *NR* |  |
| 27 | Verplancke et al. (2010)^27^ | Belgium | N | Adults >10 days | 830 | Creatinine, diuresis | 12 hours | 3 days | N | Dialysis (2-7 days) | 82 | Cross-validation (*10*-fold) | Echo-state network (recurrent neural-network in which the readout is modified using predicted and real classification data) | 0.822 (CI 0.778-0.865) | *NR* | Naïve Bayes classifier | 0.850 (0.811-0.890) | *NR* |  |
| 28 | Wang et al. (2015)^28^ | USA | Y | *NR* | 930 | Physiologic | 6 hours | 1 min | Y | Mortality (ICU) | 56 | Cross-validation (*5*-fold) | 1. Summary measures obtained (mean, median, variance, skewness and kurtosis)  2. Classification using cox regression  3. Repeated per 6 hours | 0.7514^ß^ | *NR* | Logistic regression (using whole observation trend as opposed to time-sliced trends) | 0.7463 | *NR* |  |
| 29 | Wu et al. (2017)^29^ | USA | Y | Adults >12h and <4 days w/o orders for reduced care | 15.695 | Physiologic, laboratory | 1 hour | 12 hours | Y | Specific clinical intervention (e.g. vasopressor 2h,) | 4331 | NA | 1. Switching-state autoregressive model and probability of being in positive or negative state using naïve Bayes classifier  2. Classification using these latent states and raw time series input in logistic regression | 0.92 (CI 0.92, 0.92) | *NR* | Logistic regression (cross-sectional) | 0.89 (CI 0.89, 0.89) | *NR* |  |

1. Linear predictor is reported, but not the standard deviation

2. Accuracy is reported

* matched case-control design(!)

** Physionet challenge

∂ = calibration-in-the-large!

ß = used c-statistic not specified, cave failing to account for censoring

1. Calvert JS, Price DA, Barton CW, Chettipally UK, Das R. Discharge recommendation based on a novel technique of homeostatic analysis. *Journal of the American Medical Informatics Association : JAMIA*. 2017;24(1):24-9.

2. Calvert JS, Price DA, Chettipally UK, Barton CW, Feldman MD, Hoffman JL, Jay M, Das R. A computational approach to early sepsis detection. 2016;74:69-73.

3. Cancio LC, Galvez E, Jr., Turner CE, Kypreos NG, Parker A, Holcomb JB. Base deficit and alveolar-arterial gradient during resuscitation contribute independently but modestly to the prediction of mortality after burn injury. *Journal of burn care & research : official publication of the American Burn Association*. 2006;27(3):289-96; discussion 96.

4. Chaparro JA, Giraldo BF, Caminal P, Benito S. Analysis of the respiratory pattern variability of patients in weaning process using autoregressive modeling techniques. 2011;2011:5690-3.

5. Cheng CW, Wang MD. Improving Personalized Clinical Risk Prediction Based on Causality-Based Association Rules. *ACM-BCB : the ACM Conference on Bioinformatics, Computational Biology and Biomedicine ACM Conference on Bioinformatics, Computational Biology and Biomedicine*. 2015;2015:386-92.

6. Crump C, Saxena S, Wilson B, Farrell P, Rafiq A, Silvers CT. Using Bayesian networks and rule-based trending to predict patient status in the intensive care unit. 2009;2009.

7. Cuthbertson BH, Boroujerdi M, McKie L, Aucott L, Prescott G. Can physiological variables and early warning scoring systems allow early recognition of the deteriorating surgical patient? 2007;35(2):402-9.

8. Ebadollahi S, Sun J, Gotz D, Hu J, Sow D, Neti C. Predicting Patient's Trajectory of Physiological Data using Temporal Trends in Similar Patients: A System for Near-Term Prognostics. 2010;2010:192-6.

9. Ghose S, Mitra J, Khanna S, Dowling J. An Improved Patient-Specific Mortality Risk Prediction in ICU in a Random Forest Classification Framework. *Stud Health Technol Inform*. 2015;214:56-61.

10. Ghosh S, Li J, Cao L, Ramamohanarao K. Septic shock prediction for ICU patients via coupled HMM walking on sequential contrast patterns. *Journal of Biomedical Informatics*. 2017;66:19-31.

11. Guiza F, Depreitere B, Piper I, Van den Berghe G, Meyfroidt G. Novel methods to predict increased intracranial pressure during intensive care and long-term neurologic outcome after traumatic brain injury: development and validation in a multicenter dataset. 2013;41(2):554-64.

12. Kam HJ, Kim HY. Learning representations for the early detection of sepsis with deep neural networks. 2017;89:248-55.

13. Kennedy CE, Aoki N, Mariscalco M, Turley JP. Using Time Series Analysis to Predict Cardiac Arrest in a PICU. *Pediatric critical care medicine : a journal of the Society of Critical Care Medicine and the World Federation of Pediatric Intensive and Critical Care Societies*. 2015;16(9):e332-9.

14. Last M, Tosas O, Gallo Cassarino T, Kozlakidis Z, Edgeworth J. Evolving classification of intensive care patients from event data. 2016;69:22-32.

15. Lee J, Mark R. A Hypotensive Episode Predictor for Intensive Care based on Heart Rate and Blood Pressure Time Series. *Computing in cardiology*. 2011;2010(26-29 Sept. 2010):81-4.

16. Lee J, Mark RG. An investigation of patterns in hemodynamic data indicative of impending hypotension in intensive care. 2010;9:62.

17. Levin SR, Harley ET, Fackler JC, Lehmann CU, Custer JW, France D, Zeger SL. Real-time forecasting of pediatric intensive care unit length of stay using computerized provider orders. 2012;40(11):3058-64.

18. Megjhani M, Terilli K, Frey HP, Velazquez AG, Doyle KW, Connolly ES, Roh DJ, Agarwal S, Claassen J, Elhadad N, Park S. Incorporating High-Frequency Physiologic Data Using Computational Dictionary Learning Improves Prediction of Delayed Cerebral Ischemia Compared to Existing Methods. *Frontiers in neurology*. 2018;9:122.

19. Minne L, Toma T, de Jonge E, Abu-Hanna A. Assessing and combining repeated prognosis of physicians and temporal models in the intensive care. *Artif Intell Med*. 2013;57:111-7.

20. Pereira RD, Salgado CM, Dejam A, Reti SR, Vieira SM, Sousa JM, Celi LA, Finkelstein SN. Fuzzy Modeling to Predict Severely Depressed Left Ventricular Ejection Fraction following Admission to the Intensive Care Unit Using Clinical Physiology. *Scientific World Journal*. 2015;2015:212703.

21. Rivera-Fernandez R, Castillo-Lorente E, Nap R, Vazquez-Mata G, Reis Miranda D. Relationship between mortality and first-day events index from routinely gathered physiological variables in ICU patients. 2012;36(9):634-43.

22. Saria S, Rajani AK, Gould J, Koller D, Penn AA. Integration of early physiological responses predicts later illness severity in preterm infants. 2010;2(48):48ra65.

23. Stein DM, Hu PF, Chen HH, Yang S, Stansbury LG, Scalea TM. Computational gene mapping to analyze continuous automated physiologic monitoring data in neuro-trauma intensive care. *The journal of trauma and acute care surgery*. 2012;73(2):419-24; discussion 24.

24. Timsit JF, Fosse JP, Troche G, De Lassence A, Alberti C, Garrouste-Orgeas M, Azoulay E, Chevret S, Moine P, Cohen Y. Accuracy of a composite score using daily SAPS II and LOD scores for predicting hospital mortality in ICU patients hospitalized for more than 72 h. 2001;27(6):1012-21.

25. Tjepkema-Cloostermans MC, Hofmeijer J, Beishuizen A, Hom HW, Blans MJ, Bosch FH, Van Putten MJAM. Cerebral recovery index: Reliable help for prediction of neurologic outcome after cardiac arrest. *Critical Care Medicine*. 2017;45(8):e789-e97.

26. Toma T, Bosman RJ, Siebes A, Peek N, Abu-Hanna A. Learning predictive models that use pattern discovery--a bootstrap evaluative approach applied in organ functioning sequences. 2010;43(4):578-86.

27. Verplancke T, Van Looy S, Steurbaut K, Benoit D, De Turck F, De Moor G, Decruyenaere J. A novel time series analysis approach for prediction of dialysis in critically ill patients using echo-state networks. *BMC Medical Informatics and Decision Making*. 2010;10:4.

28. Wang Y, Chen W, Heard K, Kollef MH, Bailey TC, Cui Z, He Y, Lu C, Chen Y. Mortality Prediction in ICUs Using A Novel Time-Slicing Cox Regression Method. 2015;2015:1289-95.

29. Wu M, Ghassemi M, Feng M, Celi LA, Szolovits P, Doshi-Velez F. Understanding vasopressor intervention and weaning: risk prediction in a public heterogeneous clinical time series database. *Journal of the American Medical Informatics Association : JAMIA*. 2017;24(3):488-95.
